# Association of Severe COVID-19 and Persistent COVID-19 Symptoms With Economic Hardship Among US Families

**DOI:** 10.1001/jamanetworkopen.2023.47318

**Published:** 2023-12-12

**Authors:** Nicole L. Hair, Carly Urban

**Affiliations:** 1 Department of Health Services Policy and Management, University of South Carolina Arnold School of Public Health, Columbia; 2 Department of Economics, Montana State University, Bozeman

## Abstract

**Importance:**

Little is known about the association of severe COVID-19 and post–COVID-19 conditions with household finances.

**Objective:**

To examine associations between COVID-19 outcomes, pandemic-related economic hardship, and prepandemic socioeconomic status among families in the US.

**Design, Setting, and Participants:**

This cohort study used data from the Panel Study of Income Dynamics (PSID), a nationally representative, longitudinal study. Data included 6932 families active in the PSID in both 2019 and 2021.

**Exposures:**

Ordinal exposure categories were defined based on whether the reference person or spouse or partner reported a positive COVID-19 diagnosis and (1) persistent COVID-19 symptoms, (2) previous severe COVID-19, or (3) previous moderate, mild, or asymptomatic COVID-19. Families with no history of COVID-19 served as the reference group.

**Main Outcomes and Measures:**

Outcomes included whether a resident family member was laid off or furloughed, lost earnings, or had any financial difficulties due to the COVID-19 pandemic.

**Results:**

In this cohort study of 6932 families (772 Hispanic families [weighted, 13.5%; 95% CI, 12.4%-14.6%], 2725 non-Hispanic Black families [weighted, 13.1%; 95% CI, 12.3%-14.1%], and 3242 non-Hispanic White families [weighted, 66.8%; 95% CI, 65.2%-68.3%]), close to 1 in 4 (2222 [weighted, 27.0%; 95% CI, 25.6%-28.6%]) reported income below 200% of the US Census Bureau poverty threshold. In survey-weighted regression models adjusted for prepandemic sociodemographic characteristics and experiences of economic hardship, the odds of reporting pandemic-related economic hardship were 2.0 to 3.7 times higher among families headed by an adult with persistent COVID-19 symptoms (laid off or furloughed: adjusted odds ratio [AOR], 1.98 [95% CI, 1.37-2.85]; lost earnings: AOR, 2.86 [95% CI, 2.06-3.97]; financial difficulties: AOR, 3.72 [95% CI, 2.62-5.27]) and 1.7 to 2.0 times higher among families headed by an adult with previous severe COVID-19 (laid off or furloughed: AOR, 1.69 [95% CI, 1.13-2.53]; lost earnings: AOR, 1.99 [95% CI, 1.37-2.90]; financial difficulties: AOR, 1.87 [95% CI, 1.25-2.80]) compared with families with no history of COVID-19. Families headed by an adult with persistent COVID-19 symptoms had increased odds of reporting financial difficulties due to the pandemic regardless of prepandemic socioeconomic status (families with lower income: AOR, 3.71 [95% CI, 1.94-7.10]; families with higher income: AOR, 3.74 [95% CI, 2.48-5.63]). Previous severe COVID-19 was significantly associated with financial difficulties among families with lower income (AOR, 2.59 [95% CI, 1.26-5.31]) but was not significantly associated with financial difficulties among those with high income (OR, 1.56 [95% CI, 0.95-2.56]).

**Conclusions and Relevance:**

This cohort study suggests that persistent COVID-19 symptoms and, to a lesser extent, previous severe COVID-19 were associated with increased odds of pandemic-related economic hardship in a cohort of US families. The economic consequences of COVID-19 varied according to socioeconomic status; families with lower income before the pandemic were more vulnerable to employment disruptions and earnings losses associated with an adult family member’s COVID-19 illness.

## Key Points

**Question:**

Are COVID-19 outcomes, including severe COVID-19 and persistent COVID-19 symptoms, associated with economic hardship among US families?

**Findings:**

In this cohort study of 6932 families, the odds of reporting economic hardship were higher for families headed by an adult with persistent COVID-19 symptoms and, to a lesser extent, families headed by an adult with previous severe COVID-19 compared with families with no history of COVID-19. Families with lower income before the pandemic were more vulnerable to employment disruptions and earnings losses associated with an adult family member’s COVID-19 illness.

**Meaning:**

Policy actions to mitigate the household financial hardship of post–COVID-19 conditions merit continued discussion.

## Teaser

This cohort study examines associations between COVID-19 outcomes, pandemic-related economic hardship, and prepandemic socioeconomic status among families in the US.

## Introduction

More than 5.9 million COVID-19–associated hospital admissions have been recorded among US adults.^1^ It is estimated that at least 3 million to 5 million US adults are currently living with activity-limiting post–COVID-19 conditions (PCCs) and that approximately 15% of the US adult population (approximately 39 million adults) have, at some point, experienced PCCs.^2,3^ Beyond significant personal health effects, there is accumulating evidence that patients with severe COVID-19 and PCCs face considerable economic consequences. Symptoms of PCCs can adversely affect daily functioning and have been associated with a lower likelihood of working full time and a higher likelihood of being unemployed.^4,5^ COVID-19–associated hospitalizations have been similarly associated with job loss.^6^ In addition to the direct associations with patients’ work and earnings, household finances may be further stressed by out-of-pocket medical expenses and lost caretaker productivity.^6,7,8,9^ The consequences may be even more severe for families with low income who have fewer resources available to buffer against COVID-19–related financial shocks but appear to be more likely to experience severe or long-term health effects from COVID-19.^10,11^

This study used data from the Panel Study of Income Dynamics (PSID), a long-running, nationally representative household panel survey with interviews conducted both before and during the COVID-19 pandemic, to examine associations between COVID-19 exposure and economic hardship at the family level and, furthermore, whether these associations vary according to a family’s prepandemic socioeconomic status. We hypothesized (1) that COVID-19 exposure would be positively associated with experiences of economic hardship, (2) that the strength of these associations would vary according to the duration and severity of COVID-19 symptoms, and (3) that the economic sequelae of COVID-19 would be most pronounced among families with lower income before the pandemic.

## Methods

This cohort study was deemed exempt from review by the Office of Research Compliance, an administrative office that supports the University of South Carolina Institutional Review Board, because it involved secondary analysis of publicly available, deidentified data. We followed the Strengthening the Reporting of Observational Studies in Epidemiology (STROBE) reporting guideline for cohort studies.^12^

### Data Source

We used unrestricted public use data from the PSID, a nationally representative, longitudinal study of families in the US.^13^ The core PSID survey, fielded biennially, collects detailed information on income, employment, wealth, and family composition. The PSID collects detailed information for the family’s reference person (RP) and, if present, their spouse or partner (SP). Fewer details are collected for other family members. In 2021, the PSID added questions to assess the outcome of the COVID-19 pandemic on study families, including COVID-19–related health outcomes and pandemic-related financial difficulties. The PSID-2021 survey was fielded from March 19 to December 31, 2021.

The PSID-2021 Early Release file includes data collected from a balanced panel of 8468 families who were active in the PSID in both 2019 and 2021. We merged postpandemic measures of family economic hardship and COVID-19–related health outcomes collected in the PSID-2021 survey with prepandemic sociodemographic characteristics collected in the PSID-2019 survey. We excluded families with incomplete sociodemographic or economic information (n = 259) or indeterminate COVID-19 exposure (n = 1277). The study cohort comprised 6932 families.

### Measures

#### Economic Hardship

We examined 3 indicators of family-level economic hardship reported by the RP in the PSID-2021 survey. They were (1) resident family member laid off or furloughed due to the pandemic, (2) resident family member lost earnings due to the pandemic, and (3) resident family member had financial difficulties due to the pandemic.

#### COVID-19 Exposure

Information related to COVID-19 infection and severity and duration of COVID-19 symptoms was collected for the RP and SP (if present). The RP was asked if they or their SP had “talked to a doctor or other health care professional about whether [they] may have had COVID-19.” An affirmative response was followed by, “Did they say that [you/your SP] definitely had COVID-19, probably had it, may have had it, probably did not have it, or definitely did not have COVID-19?” If the RP reported that they or their SP were told that they definitely or probably had COVID-19, they were considered to have a positive COVID-19 diagnosis. Follow-up questions ascertained whether the RP or their SP was admitted to a hospital because of COVID-19 (yes or no); had experienced any COVID-19 symptoms (yes or no) and, if so, how bad or bothersome these symptoms were at their worst (mild, moderate, severe, or very severe); or were currently experiencing lingering physical or mental health effects from COVID-19 (yes or no). Families headed by at least 1 adult (RP and/or SP) with a COVID-19 diagnosis were assigned to 1 of 3 ordinal exposure categories:

1. Persistent COVID-19: families headed by at least 1 adult who, at the time of the survey, was currently experiencing lingering health effects from COVID-19;
2. Severe COVID-19: families with no indication of persistent COVID-19 headed by at least 1 adult who was hospitalized or experienced severe or very severe symptoms due to COVID-19; and
3. Moderate, mild, or asymptomatic COVID-19: families with no indication of severe or persistent COVID-19 headed by at least 1 adult who had moderate, mild, or no symptoms associated with their previous COVID-19 illness.

If the RP reported that they or their SP had not talked to a health care professional or were told that they may, probably did not, or definitely did not have COVID-19, they were asked if they had “symptoms or exposure… that led [them] to believe [they] had COVID-19” and, when applicable, about the severity and duration of symptoms. Families with no history of COVID-19 illness (ie, no COVID-19 diagnosis and no self-reported COVID-19 symptoms or lingering health effects) were assigned to a reference group. Families with indeterminate COVID-19 exposure (eg, incomplete data on COVID-19 health outcomes or self-reported COVID-19 symptoms or lingering health effects without a corresponding COVID-19 diagnosis) were excluded.

#### Covariates

We included the following sociodemographic characteristics collected in the PSID-2019: any children younger than 16 years in the family (yes or no), age of the RP, self-reported race and ethnicity of the RP (Hispanic, non-Hispanic Black, non-Hispanic White, or other [including non-Hispanic Alaska Native, American Indian, Asian, or Pacific Islander persons or individuals who reported other race]), highest level of education completed by the RP or SP (less than college, some college, or college graduate), total family income relative to US Census Bureau poverty thresholds^14^ (income below or above 200% of the poverty threshold), whether any family member went without health insurance in the past 2 years (yes or no), geographic region (northeast, north central, south, or west), and residence in a nonmetropolitan area (yes or no). For each outcome of interest, we also identified a closely related indicator of economic hardship collected in the PSID-2019: whether the RP or SP missed work because they were temporarily laid off (yes or no), total family labor income, or whether any family member had credit card or store card debt (yes or no).

### Statistical Analysis

First, we estimated a multinomial logistic regression model to assess correlations between families’ sociodemographic characteristics and experiences of economic hardship at baseline and COVID-19 exposure. The no COVID-19 exposure category served as the reference outcome. Results are presented as adjusted relative risk ratios (ARRRs) with 95% CIs.

Next, we estimated logistic regression models to examine associations between COVID-19 exposure (persistent; severe; or moderate, mild, or asymptomatic COVID-19 vs no COVID-19) and 3 indicators of pandemic-related economic hardship (laid off or furloughed, lost earnings, and financial difficulties) among US families. Separate models were fit for each economic outcome. Results are presented as unadjusted odds ratios (ORs) or adjusted ORs (AORs) with 95% CIs. Adjusted models included a range of potentially confounding sociodemographic characteristics and, to account for the possibility that COVID-19 exposure may be associated with economic hardship at baseline, a closely related economic indicator collected in the PSID-2019 (Table 1). We further examined whether associations between COVID-19 exposure and economic hardship varied by prepandemic socioeconomic status. Differences across subgroups were evaluated by fitting separate regression models stratified by prepandemic family income (below vs above 200% of the poverty threshold).

**Table 1.** Characteristics of Families in the PSID, 2019-2021^a^

Characteristic	No., unweighted	% (95% CI), weighted
Economic hardship, reported in PSID-2021		
Laid off or furloughed		
Yes	1340	17.0 (15.9-18.3)
No	5592	83.0 (81.7-84.1)
Lost earnings		
Yes	1780	23.0 (21.7-24.4)
No	5152	77.0 (75.6-78.3)
Financial difficulties		
Yes	1489	16.8 (15.6-18.0)
No	5443	83.2 (82.0-84.4)
COVID-19 symptoms, reported in PSID-2021		
Persistent	303	4.4 (3.8-5.1)
Severe	243	3.1 (2.6-3.7)
Moderate, mild, or asymptomatic	601	7.9 (7.2-8.8)
No history	5785	84.6 (83.4-85.7)
Sociodemographic characteristics, reported in PSID-2019		
Children younger than 16 y in family		
Yes	2388	23.2 (22.0-24.5)
No	4544	76.8 (75.5-78.0)
Age of RP, mean (SD), y	52.8 (17.5)	NA
Race and ethnicity of RP		
Hispanic	772	13.5 (12.4-14.6)
Non-Hispanic Black	2725	13.1 (12.3-14.1)
Non-Hispanic White	3242	66.8 (65.2-68.3)
Other^b^	193	6.7 (5.7-7.8)
Highest level of education of RP or SP		
Less than college	2069	29.6 (28.0-31.1)
Some college	2577	33.6 (32.1-35.2)
College graduate	2286	36.8 (35.3-38.4)
Total family income		
Below 200% of poverty threshold	2222	27.0 (25.6-28.6)
Above 200% of poverty threshold	4710	73.0 (71.4-74.4)
Any family member without health insurance		
Yes	1218	14.2 (13.1-15.4)
No	5714	85.8 (84.6-86.9)
Region		
Northeast	837	17.6 (16.3-18.9)
North central	1660	21.7 (20.4-23.0)
South	3221	38.0 (36.4-39.6)
West	1214	22.8 (21.4-24.2)
Nonmetropolitan area		
Yes	1040	15.8 (14.7-17.0)
No	5892	84.2 (83.0-85.3)
Economic hardship, reported in PSID-2019		
Laid off or furloughed		
Yes	128	1.6 (1.2-2.0)
No	6804	98.4 (98.0-98.8)
Total family labor income, mean (SD), $	63 373 (90 593)	NA
Credit card debt		
Yes	2252	35.0 (33.4-36.6)
No	4680	65.0 (63.4-66.6)

Abbreviations: NA, not applicable; PSID, Panel Study of Income Dynamics; RP, reference person; SP, spouse or partner.

^a^ The PSID-2021 Early Release file includes data collected from a balanced panel of 8468 families who were active in the PSID in both 2019 and 2021. We excluded families with incomplete sociodemographic information (n = 259) or indeterminate COVID-19 exposure (n = 1277). The study cohort comprised 6932 families.

^b^ Includes non-Hispanic Alaska Native, American Indian, Asian, or Pacific Islander persons and individuals who reported other race.

All analyses were conducted using the survey commands in Stata, version 16 (StataCorp LLC) to apply family weights provided by the PSID. Estimates were considered statistically significant if 95% CIs did not include 1 (equivalent to 2-sided *P* < .05).

## Results

Table 1 presents unweighted frequencies and weighted percentages for sample characteristics. The study cohort comprised 6932 families. Of these, 1340 (17.0% [95% CI, 15.9%-18.3%]), 1780 (23.0% [95% CI, 21.7%-24.4%]), and 1489 (16.8% [95% CI, 15.6%-18.0%]) reported that a resident family member had been laid off or furloughed, lost earnings, or had financial difficulties, respectively, due to the COVID-19 pandemic. A total of 15.4% (95% CI, 14.3%-16.5%) of families were headed by an adult (RP and/or SP) with a COVID-19 diagnosis, including 303 (4.4% [95% CI, 3.8%-5.1%]), 243 (3.1% [95% CI, 2.6%-3.7%]), and 601 (7.9% [95% CI, 7.2%-8.8%]) with a history of persistent COVID-19, severe COVID-19, or moderate, mild, or asymptomatic COVID-19, respectively. Close to 1 in 4 households (28.4% [95% CI, 24.8%-32.1%]) headed by an adult with a prior COVID-19 diagnosis reported symptoms consistent with PCCs. The remaining 5785 in-sample families (84.6% [95% CI, 83.4%-85.7%]) had no history of COVID-19 illness.

The sample was racially and ethnically diverse; 772 families (13.5% [95% CI, 12.4%-14.6%]) were headed by a Hispanic RP, 2725 (13.1% [95% CI, 12.3%-14.1%]) by a non-Hispanic Black RP, and 3242 (66.8% [95% CI, 65.2%-68.3%]) by a non-Hispanic White RP (Table 1). Nearly 25% of families (2388 families [23.2%; 95% CI, 22.0%-24.5%]) included at least 1 resident child younger than 16 years. Close to 75% of families (4863 families [70.4%; 95% CI, 68.9%-72.0%]) were headed by an adult (RP or SP) with at least some college education, including 2286 families (36.8% [95% CI, 35.3%-38.4%]) headed by a college graduate. Close to 1 in 4 families (2222 families [27.0%; 95% CI, 25.6%-28.6%]) reported total family income below 200% of the poverty threshold; 1218 families (14.2% [95% CI, 13.1%-15.4%]) had at least 1 member who went without health insurance in the past 2 years, and 1040 (15.8% [95% CI, 14.7%-17.0%]) resided in a nonmetropolitan area.

Table 2 presents results from a multinomial logistic regression in which the reference outcome is no COVID-19 exposure. Accounting for baseline family characteristics, prepandemic experiences of economic hardship were associated with COVID-19 exposure. Families headed by an adult who missed work because they were temporarily laid off or furloughed were at higher risk of persistent COVID-19 symptoms (ARRR, 2.82 [95% CI, 1.34-5.93]), while families with credit card or store card debt (an indicator of financial difficulties) were at higher risk of moderate, mild, or asymptomatic COVID-19 infection (ARRR, 1.58 [95% CI, 1.25-2.00]).

**Table 2.** Associations Between Baseline Family Characteristics and COVID-19 Exposure

Characteristic	ARRR (95% CI)^a^		
	Persistent COVID-19	Severe COVID-19	Moderate, mild, or asymptomatic COVID-19
Sociodemographic characteristics			
Children younger than 16 y in family			
Yes	1.66 (1.19-2.30)	1.40 (0.94-2.08)	1.54 (1.19-2.00)
No	1 [Reference]	1 [Reference]	1 [Reference]
Age of RP, mean (SD), y	1.00 (0.99-1.01)	0.99 (0.98-1.00)	0.98 (0.97-0.99)
Race and ethnicity of RP			
Hispanic	1.42 (0.58-3.45)	2.16 (0.73-6.39)	4.76 (1.62-13.96)
Non-Hispanic Black	0.78 (0.32-1.93)	0.84 (0.28-2.53)	2.52 (0.87-7.30)
Non-Hispanic White	0.86 (0.37-1.98)	0.93 (0.32-2.68)	3.05 (1.08-8.65)
Other^b^	1 [Reference]	1 [Reference]	1 [Reference]
Highest level of education of RP or SP			
Less than college	1 [Reference]	1 [Reference]	1 [Reference]
Some college	1.57 (1.03-2.39)	1.20 (0.77-1.86)	0.93 (0.66-1.29)
College graduate	1.00 (0.62-1.61)	0.75 (0.43-1.28)	1.28 (0.90-1.82)
Total family income			
Below 200% of poverty threshold	0.97 (0.64-1.47)	1.13 (0.72-1.77)	0.60 (0.44-0.83)
Above 200% of poverty threshold	1 [Reference]	1 [Reference]	1 [Reference]
Any family member without health insurance			
Yes	1.21 (0.81-1.80)	1.03 (0.64-1.65)	1.06 (0.76-1.49)
No	1 [Reference]	1 [Reference]	1 [Reference]
Region			
Northeast	1 [Reference]	1 [Reference]	1 [Reference]
North central	1.09 (0.65-1.84)	1.46 (0.77-2.79)	1.20 (0.80-1.79)
South	1.03 (0.62-1.71)	1.03 (0.55-1.93)	1.09 (0.74-1.60)
West	1.08 (0.63-1.85)	1.19 (0.59-2.38)	0.93 (0.61-1.41)
Nonmetropolitan area			
Yes	1.24 (0.81-1.88)	0.95 (0.56-1.62)	1.38 (1.02-1.88)
No	1 [Reference]	1 [Reference]	1 [Reference]
Economic hardship			
Laid off or furloughed			
Yes	2.82 (1.34-5.93)	0.60 (0.13-2.80)	0.53 (0.23-1.21)
No	1 [Reference]	1 [Reference]	1 [Reference]
Total family labor income, $ (thousands)	1.00 (0.98-1.02)	1.01 (0.99-1.04)	1.00 (0.99-1.01)
Credit card debt			
Yes	1.34 (0.98-1.84)	1.45 (0.99-2.11)	1.58 (1.25-2.00)
No	1 [Reference]	1 [Reference]	1 [Reference]

Abbreviations: ARRR, adjusted relative risk ratio; RP, reference person; SP, spouse or partner.

^a^ Results from multinomial logistic regression including all COVID-19 exposure groups, with the no COVID-19 group used as the comparator. Baseline sociodemographic characteristics and indicators of economic hardship were reported in the Panel Study of Income Dynamics–2019 survey (prior to the pandemic). The sample includes 6932 families.

^b^ Includes non-Hispanic Alaska Native, American Indian, Asian, or Pacific Islander persons and individuals who reported other race.

Table 3 presents results from unadjusted and adjusted logistic regression models. Compared with families with no history of COVID-19, families headed by an adult with persistent COVID-19 symptoms had at least a 2-fold greater odds of having a resident family member who was laid off or furloughed (OR, 2.31 [95% CI, 1.64-3.25]; AOR, 1.98 [95% CI, 1.37-2.85]) or lost earnings (OR, 3.07 [95% CI, 2.24-4.21]; AOR, 2.86 [95% CI, 2.06-3.97]) and a more than 3-fold greater odds of reporting any financial difficulties (OR, 3.82 [95% CI, 2.77-5.27]; AOR, 3.72 [95% CI, 2.62-5.27]) due to the pandemic. Associations between acute COVID-19 symptoms (ie, COVID-19 symptoms resolved by the time of the PSID-2021 survey) and indicators of economic hardship were generally less strong but varied significantly by symptom severity. The odds of a resident family member being laid off or furloughed (OR, 2.03 [95% CI, 1.35-3.05]; AOR, 1.69 [95% CI, 1.13-2.53]), losing earnings (OR, 2.30 [95% CI, 1.57-3.36]; AOR, 1.99 [95% CI, 1.37-2.90]), or having financial difficulties (OR, 2.28 [95% CI, 1.53-3.39]; AOR, 1.87 [95% CI, 1.25-2.80]) due to the pandemic were 1.7 to 2.0 times greater among families headed by an adult with previous severe COVID-19 compared with families with no history of COVID-19. In unadjusted models, a history of moderate, mild, or asymptomatic COVID-19 infection was associated with greater odds of a resident family member losing earnings due to the pandemic (OR, 1.41 [95% CI, 1.09-1.82]); however, the association was no longer statistically significant after adjusting for baseline family characteristics (AOR, 1.21 [95% CI, 0.92-1.58]). We found no statistically significant association between previous moderate, mild, or asymptomatic COVID-19 and a resident family member either being laid off or furloughed (OR, 1.22 [95% CI, 0.91-1.62]; AOR, 1.05 [95% CI, 0.78-1.42]) or experiencing financial difficulties (OR, 1.05 [95% CI, 0.78-1.40]; AOR, 0.91 [95% CI, 0.67-1.24]) due to the pandemic. In sensitivity analyses, results were similar when we considered only those who were told by a health care professional that they definitely had COVID-19 as having a COVID-19 diagnosis (eTable in Supplement 1).

**Table 3.** Unadjusted ORs and AORs of Family Economic Hardship by COVID-19 Exposure^a^

Exposure	Laid off or furloughed	Lost earnings	Financial difficulties
**Unadjusted models, OR (95% CI)**			
Persistent COVID-19	2.31 (1.64-3.25)	3.07 (2.24-4.21)	3.82 (2.77-5.27)
Severe COVID-19	2.03 (1.35-3.05)	2.30 (1.57-3.36)	2.28 (1.53-3.39)
Moderate, mild, or asymptomatic COVID-19	1.22 (0.91-1.62)	1.41 (1.09-1.82)	1.05 (0.78-1.40)
No COVID-19	1 [Reference]	1 [Reference]	1 [Reference]
**Adjusted models, AOR (95% CI)** ^b^			
Persistent COVID-19	1.98 (1.37-2.85)	2.86 (2.06-3.97)	3.72 (2.62-5.27)
Severe COVID-19	1.69 (1.13-2.53)	1.99 (1.37-2.90)	1.87 (1.25-2.80)
Moderate, mild, or asymptomatic COVID-19	1.05 (0.78-1.42)	1.21 (0.92-1.58)	0.91 (0.67-1.24)
No COVID-19	1 [Reference]	1 [Reference]	1 [Reference]

Abbreviations: AOR, adjusted odds ratio; OR, odds ratio.

^a^ Sample includes 6932 families.

^b^ All adjusted models include controls for resident children, age of the reference person (RP), race and ethnicity of the RP, highest level of education completed by the RP or spouse or partner (SP), total family income relative to poverty thresholds, uninsurance, geographic region, and residence in a nonmetropolitan area. In addition, to account for potential differences in baseline economic hardship, each adjusted model includes a similar economic indicator collected in the Panel Study of Income Dynamics–2019 survey as a covariate: whether the RP or SP missed work because they were temporarily laid off, total family labor income, or whether anyone in the family had credit card or store card debt.

Table 4 presents results separately for families with a prepandemic income below or above 200% of the poverty threshold. Among families with lower income, the odds of experiencing economic hardship due to the pandemic were 2.5 to 4.9 times greater for families headed by an adult with persistent COVID-19 symptoms (laid off or furloughed: AOR, 2.46 [95% CI, 1.25-5.28]; lost earnings: AOR, 4.88 [95% CI, 2.62-9.11]; financial difficulties: AOR, 3.71 [95% CI, 1.94-7.10]) or with previous severe COVID-19 (laid off or furloughed: AOR, 2.76 [95% CI, 1.29-5.90]; lost earnings: AOR, 4.61 [95% CI, 2.34-9.06]; financial difficulties: AOR, 2.59 [95% CI, 1.26-5.31]) than for families with no history of COVID-19. In addition, compared with families with lower income and no history of COVID-19, families with lower income headed by an adult with previous moderate, mild, or asymptomatic COVID-19 had a close to 2-fold greater odds of having a resident family member who lost earnings (AOR, 1.86 [95% CI, 1.02-3.39]) due to the pandemic; positive associations with a family member being laid off or furloughed (AOR, 1.68 [95% CI, 0.91-3.07]) or experiencing financial difficulties (AOR, 1.06 [95% CI, 0.61-1.84]) were not statistically significant.

**Table 4.** AORs of Family Economic Hardship by COVID-19 Exposure and Prepandemic Income^a^

Exposure	AOR (95% CI)^b^		
	Laid off or furloughed	Lost earnings	Financial difficulties
**Below 200% of poverty threshold** ^c^			
Persistent COVID-19	2.46 (1.25-5.28)	4.88 (2.62-9.11)	3.71 (1.94-7.10)
Severe COVID-19	2.76 (1.29-5.90)	4.61 (2.34-9.06)	2.59 (1.26-5.31)
Moderate, mild, or asymptomatic COVID-19	1.68 (0.91-3.07)	1.86 (1.02-3.39)	1.06 (0.61-1.84)
No COVID-19	1 [Reference]	1 [Reference]	1 [Reference]
**Above 200% of poverty threshold** ^d^			
Persistent COVID-19	1.81 (1.19-2.75)	2.37 (1.62-3.46)	3.74 (2.48-5.63)
Severe COVID-19	1.36 (0.84-2.19)	1.44 (0.93-2.24)	1.56 (0.95-2.56)
Moderate, mild, or asymptomatic COVID-19	0.92 (0.65-1.30)	1.10 (0.81-1.50)	0.85 (0.58-1.25)
No COVID-19	1 [Reference]	1 [Reference]	1 [Reference]

Abbreviation: AOR, adjusted odds ratio.

^a^ Differences across subgroups were evaluated by fitting separate regression models stratified by total family income (above vs below 200% of the US Census Bureau poverty threshold) reported in the Panel Study of Income Dynamics–2019 survey.

^b^ All adjusted models include controls for resident children, age of the reference person (RP), race and ethnicity of the RP, highest level of education completed by the RP or spouse or partner (SP), total family income relative to poverty thresholds, uninsurance, geographic region, and residence in a nonmetropolitan area. In addition, to account for potential differences in baseline economic hardship, each adjusted model includes a similar economic indicator collected in the Panel Study of Income Dynamics–2019 survey as a covariate: whether the RP or SP missed work because they were temporarily laid off, total family labor income, or whether anyone in the family had credit card or store card debt.

^c^ Sample includes 2222 families with total family income below 200% of the US Census Bureau poverty threshold.

^d^ Sample includes 4710 families with total family income above 200% of the US Census Bureau poverty threshold.

Among families with higher income, the odds of a resident member being laid off or furloughed (AOR, 1.81 [95% CI, 1.19-2.75]), losing earnings (AOR, 2.37 [95% CI, 1.62-3.46]), or having financial difficulties (AOR, 3.74 [95% CI, 2.48-5.63]) due to the pandemic were 1.8 to 3.7 times greater for families headed by an adult with persistent COVID-19 symptoms than for families with no COVID-19 (Table 4). Positive associations between previous severe COVID-19 and pandemic-related economic hardship (laid off or furloughed: AOR, 1.36 [95% CI, 0.84-2.19]; lost earnings: AOR, 1.44 [95% CI, 0.93-2.24]; financial difficulties: AOR, 1.56 [95% CI, 0.95-2.56]) were not statistically significant among families with higher income. We found no statistically significant associations between previous moderate, mild, or asymptomatic COVID-19 and experiences of economic hardship (laid off or furloughed: AOR, 0.92 [95% CI, 0.65-1.30]; lost earnings: AOR, 1.10 [95% CI, 0.81-1.50]; financial difficulties: AOR, 0.85 [95% CI, 0.58-1.25]) among families with higher income.

## Discussion

This cohort study contributes timely, policy-relevant information on how COVID-19 and PCCs may be associated with families’ economic well-being. Using data from a long-running, nationally representative household panel survey with interviews conducted both before and during the COVID-19 pandemic, we found that persistent COVID-19 symptoms and, to a lesser extent, previous (resolved) severe COVID-19 were associated with increased odds of pandemic-related economic hardship among US families. In regression models adjusted for prepandemic sociodemographic characteristics and experiences of economic hardship, the odds of a resident family member being laid off or furloughed, losing earnings, or having financial difficulties were 2.0 to 3.7 times higher among families headed by an adult with persistent COVID-19 symptoms and 1.7 to 2.0 times higher among families headed by an adult with previous severe COVID-19 compared with families with no history of COVID-19. We found no statistically significant association in the overall sample between moderate, mild, or asymptomatic COVID-19 and indicators of family economic hardship.

Compared with similar families with no history of COVID-19, families headed by an adult with persistent COVID-19 symptoms had increased odds of experiencing economic hardship regardless of their prepandemic financial status. In contrast, an adult family member’s severe COVID-19 illness was more strongly associated with indicators of economic hardship among families with lower income. On balance, families with lower income before the pandemic (ie, families who had fewer resources available to buffer against COVID-19–related financial shocks) were more vulnerable to employment disruptions and earnings losses associated with an adult family member’s COVID-19 illness. Individuals living in economically vulnerable households are more likely to hold essential jobs, which have been associated with increased employment-related exposure and risk of severe COVID-19 among workers and their household members.^15,16^ Low-wage essential workers, who typically do not have the ability to work from home, are also least likely to have access to paid leave.^17,18^ Our results highlight the extent to which the economic sequelae of COVID-19 vary according to socioeconomic status and, consequently, may compound preexisting inequalities.

A total of 15.4% of households in our sample were headed by an adult with a prior COVID-19 diagnosis. Of those, close to 1 in 4 (weighted 28.4%; 4.4% of the overall sample) reported persistent symptoms consistent with PCCs. Although point prevalence estimates of PCCs vary across studies, populations, and settings, our findings are in line with prior work that estimates 13% to 30% of those who have had COVID-19 are currently experiencing PCCs or associated conditions.^2,19,20,21^ At least 3 million to 5 million US adults are currently living with activity-limiting PCCs.^3^ Given the significant economic consequences of COVID-19 and the expectation that PCCs will continue to affect individuals and their families over the long term, policy actions to mitigate the household financial impact of PCCs (eg, expanded paid sick leave, improved workplace accommodations, and wider access to disability insurance) merit continued discussion.^22,23,24^

### Limitations

This study has some limitations. First, we cannot definitively rule out the possibility that the increased odds of economic hardship we observed among families headed by an adult with previous severe COVID-19 or persistent COVID-19 symptoms were associated with factors other than COVID-19 illness. However, leveraging the longitudinal nature of PSID data, we were able to adjust for a range of potentially confounding factors measured prior to the pandemic. The absence of a strong association between economic hardship and previous moderate, mild, or asymptomatic COVID-19 further strengthens our interpretation of the study findings. Second, measures of economic hardship, COVID-19 diagnosis, and severity and duration of COVID-19 symptoms were self-reported and thus subject to recall and misclassification bias. Third, we excluded those with possible symptoms of COVID-19 who did not talk to a health care professional. Fourth, the PSID-2021 survey was fielded from March to December 2021, so our results may not reflect later phases of the pandemic.

## Conclusions

This cohort study suggests that persistent COVID-19 symptoms and, to a lesser extent, severe COVID-19 were associated with increased odds of pandemic-related economic hardship among a cohort of US families. The economic consequences of COVID-19 varied according to socioeconomic status; families with lower income before the pandemic were more vulnerable to employment disruptions and earnings losses associated with an adult family member’s COVID-19 illness.

## Notes

Supplement 1. **eTable.** Unadjusted and Adjusted Odds Ratios of Family Economic Hardship by COVID-19 Exposure, Restricting COVID-19 Diagnoses to Those Told They “Definitely had COVID-19”

Supplement 2.
Data Sharing Statement

## References

[1] Centers for Disease Control and Prevention. COVID data tracker. Accessed July 7, 2023. https://covid.cdc.gov/covid-data-tracker

[2] National Center for Health Statistics, Centers for Disease Control and Prevention. Long COVID: household pulse survey. Accessed July 6, 2023. https://www.cdc.gov/nchs/covid19/pulse/long-covid.htm

[3] Tenforde MW, Devine OJ, Reese HE, . Point prevalence estimates of activity-limiting long-term symptoms among United States adults ≥1 month after reported severe acute respiratory syndrome coronavirus 2 infection, 1 November 2021. J Infect Dis. 2023;227(7):855-863. doi:10.1093/infdis/jiac281 35776165 PMC9278232

[4] Davis HE, Assaf GS, McCorkell L, . Characterizing long COVID in an international cohort: 7 months of symptoms and their impact. EClinicalMedicine. 2021;38:101019. doi:10.1016/j.eclinm.2021.101019 34308300 PMC8280690

[5] Perlis RH, Lunz Trujillo K, Safarpour A, . Association of post–COVID-19 condition symptoms and employment status. JAMA Netw Open. 2023;6(2):e2256152. doi:10.1001/jamanetworkopen.2022.56152 36790806 PMC9932847

[6] Admon AJ, Iwashyna TJ, Kamphuis LA, ; National Heart, Lung, and Blood Institute PETAL Network. Assessment of symptom, disability, and financial trajectories in patients hospitalized for COVID-19 at 6 months. JAMA Netw Open. 2023;6(2):e2255795. doi:10.1001/jamanetworkopen.2022.55795 36787143 PMC9929698

[7] Chua KP, Conti RM, Becker NV. Assessment of out-of-pocket spending for COVID-19 hospitalizations in the US in 2020. JAMA Netw Open. 2021;4(10):e2129894. doi:10.1001/jamanetworkopen.2021.29894 34661662 PMC8524307

[8] Chua KP, Conti RM, Becker NV. Trends in and factors associated with out-of-pocket spending for COVID-19 hospitalizations from March 2020 to March 2021. JAMA Netw Open. 2022;5(2):e2148237. doi:10.1001/jamanetworkopen.2021.48237 35157059 PMC8845007

[9] Amass T, Van Scoy LJ, Hua M, . Stress-related disorders of family members of patients admitted to the intensive care unit with COVID-19. JAMA Intern Med. 2022;182(6):624-633. doi:10.1001/jamainternmed.2022.1118 35467698 PMC9039825

[10] Subramanian A, Nirantharakumar K, Hughes S, . Symptoms and risk factors for long COVID in non-hospitalized adults. Nat Med. 2022;28(8):1706-1714. doi:10.1038/s41591-022-01909-w 35879616 PMC9388369

[11] Tan AX, Hinman JA, Abdel Magid HS, Nelson LM, Odden MC. Association between income inequality and county-level COVID-19 cases and deaths in the US. JAMA Netw Open. 2021;4(5):e218799. doi:10.1001/jamanetworkopen.2021.8799 33938935 PMC8094008

[12] von Elm E, Altman DG, Egger M, Pocock SJ, Gøtzsche PC, Vandenbroucke JP; STROBE Initiative. The Strengthening the Reporting of Observational Studies in Epidemiology (STROBE) statement: guidelines for reporting observational studies. Ann Intern Med. 2007;147(8):573-577. doi:10.7326/0003-4819-147-8-200710160-00010 17938396

[13] Panel Study of Income Dynamics: public use dataset. 2022. Institute for Social Research, University of Michigan, Ann Arbor. Accessed December 7, 2022. https://psidonline.isr.umich.edu/

[14] US Census Bureau. Poverty thresholds. Accessed July 6, 2023. https://www.census.gov/data/tables/time-series/demo/income-poverty/historical-poverty-thresholds.html

[15] McCormack G, Avery C, Spitzer AKL, Chandra A. Economic vulnerability of households with essential workers. JAMA. 2020;324(4):388-390. doi:10.1001/jama.2020.11366 32556217 PMC7303901

[16] Selden TM, Berdahl TA. Risk of severe COVID-19 among workers and their household members. JAMA Intern Med. 2021;181(1):120-122. doi:10.1001/jamainternmed.2020.6249 33165502 PMC7653534

[17] Wolfe R, Harknett K, Schneider D. Inequalities at work and the toll of COVID-19. Health Affairs. June 4, 2021. Accessed October 31, 2023. https://www.healthaffairs.org/do/10.1377/hpb20210428.863621/

[18] Boyens C, Karpman M, Smalligan J. Access to paid leave is lowest among workers with the greatest needs. Urban Institute. July 14, 2022. Accessed July 6, 2023. https://www.urban.org/research/publication/access-paid-leave-lowest-among-workers-greatest-needs

[19] Bull-Otterson L. Post–COVID conditions among adult COVID-19 survivors aged 18–64 and ≥65 years—United States, March 2020–November 2021. MMWR Morb Mortal Wkly Rep. 2022;71. doi:10.15585/mmwr.mm7121e1 PMC9368731 35925799

[20] Logue JK, Franko NM, McCulloch DJ, . Sequelae in adults at 6 months after COVID-19 infection. JAMA Netw Open. 2021;4(2):e210830. doi:10.1001/jamanetworkopen.2021.0830 33606031 PMC7896197

[21] Perlis RH, Santillana M, Ognyanova K, . Prevalence and correlates of long COVID symptoms among US adults. JAMA Netw Open. 2022;5(10):e2238804. doi:10.1001/jamanetworkopen.2022.38804 36301542 PMC9614581

[22] Levine RL. Addressing the long-term effects of COVID-19. JAMA. 2022;328(9):823-824. doi:10.1001/jama.2022.14089 35921084

[23] Cutler DM. The costs of long COVID. JAMA Health Forum. 2022;3(5):e221809. doi:10.1001/jamahealthforum.2022.1809 36219031

[24] Bach K. Is “long Covid” worsening the labor shortage? The Brookings Institution. January 11, 2022. Accessed June 20, 2023. https://www.brookings.edu/research/is-long-covid-worsening-the-labor-shortage/

