# Supplementary material for: Association of Severe COVID-19 and Persistent COVID-19 Symptoms With Economic Hardship Among US Families
Source: JAMA Netw Open. 2023 Dec 12;6(12):e2347318. doi: 10.1001/jamanetworkopen.2023.47318 (PMC10716716; doi:10.1001/jamanetworkopen.2023.47318)
Supplement: Supplement 1. — eTable. Unadjusted and Adjusted Odds Ratios of Family Economic Hardship by COVID-19 Exposure, Restricting COVID-19 Diagnoses to Those Told They “Definitely had COVID-19” [file jamanetwopen-e2347318-s001.pdf]

Supplementary Online Content

Hair NL, Urban C. Association of severe COVID-19 and persistent COVID-19 symptoms with economic hardship among US families. *JAMA Netw Open.* 2023;6(12):e2347318. doi:10.1001/jamanetworkopen.2023.47318

eTable. Unadjusted and Adjusted Odds Ratios of Family Economic Hardship by COVID-19 Exposure, Restricting COVID-19 Diagnoses to Those Told They "Definitely had COVID-19"

This supplementary material has been provided by the authors to give readers additional information about their work.

© 2023 Hair NL et al. *JAMA Network Open.*

eTable. Unadjusted and Adjusted Odds Ratios of Family Economic Hardship by COVID-19 Exposure, Restricting COVID-19 Diagnoses to Those Told They “Definitely had COVID-19”[a]

	Laid off or furloughed	Lost earnings	Financial difficulties
**Unadjusted Models**	OR (95% CI)	OR (95% CI)	OR (95% CI)
Persistent COVID-19	2.32 (1.63, 3.30)	2.95 (2.12, 4.09)	3.73 (2.67, 5.22)
Severe COVID-19	1.97 (1.26, 3.08)	2.18 (1.44, 3.30)	2.13 (1.39, 3.27)
Moderate, mild or asymptomatic COVID-19	1.18 (0.87, 1.62)	1.30 (0.98, 1.72)	1.03 (0.74, 1.42)
No COVID-19	1 [Reference]	1 [Reference]	1 [Reference]
**Adjusted Models**[b]	AOR (95% CI)	AOR (95% CI)	AOR (95% CI)
Persistent COVID-19	2.00 (1.37, 2.92)	2.73 (1.94, 3.85)	3.58 (2.48, 5.18)
Severe COVID-19	1.64 (1.06, 2.56)	1.85 (1.21, 2.83)	1.79 (1.15, 2.77)
Moderate, mild or asymptomatic COVID-19	1.03 (0.74, 1.43)	1.12 (0.84, 1.51)	0.90 (0.64, 1.27)
No COVID-19	1 [Reference]	1 [Reference]	1 [Reference]

[a] If the RP reported that [they/their SP] were told that they “definitely” had COVID-19, they were considered to have a positive COVID-19 diagnosis. Families with no history of COVID-19 illness (i.e., no COVID-19 diagnosis and no self-reported COVID-19 symptoms or lingering health effects) were assigned to a reference group. Families with indeterminant COVID-19 exposure (e.g., incomplete data on COVID-19 health outcomes or self-reported COVID-19 symptoms or lingering health effects without a corresponding COVID-19 diagnosis) were excluded. The sample includes 6784 families.

[b] All adjusted models include controls for resident children, age of the RP, race and ethnicity of the RP, highest level of education completed by the RP/SP, total family income relative to poverty thresholds, uninsurance, geographic region, and residence in a non-metropolitan area. In addition, to account for potential differences in baseline economic hardship, each adjusted model includes a similar economic indicator collected in the PSID-2019 survey as a covariate: whether the RP/SP missed work because they were temporarily laid off, total family labor income, or whether anyone in the family had credit card or store card debt.

© 2023 Hair NL et al. *JAMA Network Open.*
