# Supplementary material for: Association of Severe COVID-19 and Persistent COVID-19 Symptoms With Economic Hardship Among US Families
Source: JAMA Netw Open. 2023 Dec 12;6(12):e2347318. doi: 10.1001/jamanetworkopen.2023.47318 (PMC10716716; doi:10.1001/jamanetworkopen.2023.47318)
Supplement: Supplement 2. — Data Sharing Statement [file jamanetwopen-e2347318-s002.pdf]

Data Sharing Statement

Hair. Association of Severe COVID-19 and Persistent COVID-19 Symptoms With Economic Hardship Among US Families. *JAMA Netw Open*. Published December 12, 2023. doi:10.1001/jamanetworkopen.2023.47318

Data
Data available: Yes
Data types: Deidentified participant data, Data dictionary
How to access data: https://www.openicpsr.org/openicpsr/psid
When available: With publication

Supporting Documents
Document types: None

Additional Information
Who can access the data: Anyone requesting the data who have agreed to the conditions of use.
Types of analyses: Any purpose.
Mechanisms of data availability: With signed data access agreement through Open ICPSR.
